# Effectiveness of Pyrethroid-Piperonyl Butoxide Nets Versus Standard Pyrethroid-Only Nets in Preventing Malaria in Children Under 10 Years Living in Kisantu Health Zone, Democratic Republic of the Congo

**DOI:** 10.3390/tropicalmed10060172

**Published:** 2025-06-18

**Authors:** Gillon Ilombe, Joris Losimba Likwela, Philippe Lukanu, Aimée Lulebo, Nicole Muela, Joachim Mariën, Kennedy Makola Mbanzulu, Baby Mabanzila, Junior Rika Matangila, Fiacre Agossa, Eric Mukomena, Sylvie Linsuke, Albert Kalonji, Pascal Lutumba, Jean-Pierre Van Geertruyden, Seth R. Irish

**Affiliations:** 1 National Malaria Control Program (PNLP), Ministry of Health, 3040 Kinshasa I, Democratic Republic of the Congo; erictropmed05@gmail.com; 2 Unit of Clinical Pharmacology and Pharmacovigilance, University of Kinshasa, 212 Kinshasa XI, Democratic Republic of the Congo; 3 Global Health Institute, Faculty of Medicine, University of Antwerp, 2000 Antwerp, Belgium; sylvie_lin2003@yahoo.fr (S.L.); jean-pierre.vangeertruyden@uantwerpen.be (J.-P.V.G.); 4 SANRU Asbl-Primary Health Care in Rural Areas, Kinshasa 01015, Democratic Republic of the Congo; joris.likwela@sanru.org (J.L.L.); philippe.lukanu@sanru.org (P.L.); albert.kalonji@sanru.org (A.K.); 5 Public Health School, Faculty of Medicine, University of Kinshasa, 11850 Kinshasa I, Democratic Republic of the Congo; aimelulebo@yahoo.fr; 6 Programme Elargi de Vaccination (PEV), Ministry of Public Health, 3040 Kinshasa I, Democratic Republic of the Congo; muelailombe@gmail.com; 7 Evolutionary Ecology Group, University of Antwerp, 2000 Antwerp, Belgium; joachim.marien@uantwerpen.be; 8 Department of Tropical Medicine, University of Kinshasa, 747 Kinshasa XI, Democratic Republic of the Congo; mbanzulu.kennedy@sacids.org (K.M.M.); babymabanzila@gmail.com (B.M.); junior.matangila@sacids.org (J.R.M.); pascal_lutumba@yahoo.fr (P.L.); 9 PMI VectorLink Project, Abt Associates Inc., 6130 Executive Blvd, Rockville, MD 20852, USA; rofargossa@yahoo.fr; 10 Department of Epidemiology, National Institute of Biomedical Research (INRB), 1197 Kinshasa I, Democratic Republic of the Congo; 11 Global Malaria Programme (GMP), World Health Organization (WHO), 1211 Genève, Switzerland; irishs@who.int

**Keywords:** *Plasmodium* infection, pyrethroid-piperonyl butoxide nets, preventing malaria, Kisantu Health Zone, the Democratic Republic of the Congo

## Abstract

Democratic Republic of the Congo (DRC) is among the countries that have a high malaria incidence. In an effort to combat this public health challenge, innovative tools and strategies are being developed and evaluated. Among the new generation of nets with improved effectiveness of insecticides, those treated with a combination of piperonyl butoxide (PBO) and pyrethroids appear to be a promising malaria control tool. This study evaluated the effectiveness of this combination under community conditions of use in the DRC. A quasi-experimental study was carried out from January to December 2018, in Kisantu Health Zone. Thirty villages were randomly allocated as clusters (1:1) to receive one of two types of long-lasting insecticidal nets (LLIN) treated with deltamethrin alone, or PBO with deltamethrin. After the intervention, the assessments were conducted monthly, quarterly, and every six months for malaria infection, mosquito density, and LLIN durability, respectively. Comparison of changes in different indices between the two groups was made using generalized linear models to correct for non-linear effects. A total of 1790 children were included. There was a significant non-linear effect of time (months) on the malaria infection incidence. The malaria infection incidence was higher in January–March, May–June, and November. It remained higher in the control group compared to the intervention group over time. Similarly, there was a significant non-linear effect of time on the density of both *Anopheles funestus* s.l. and *Anopheles gambiae* s.l. These densities decreased after the first month following the intervention and increased after 6 months. Twelve months later, a cohort follow-up showed that the bio-efficacy of LLINs was better in the intervention group. The nets treated with the combination of PBO and deltamethrin appear to be more effective for malaria control under community conditions in the DRC, but a loss of chemical durability is noted after the first year of use.

## 1. Introduction

Malaria remains a major burden in the Democratic Republic of the Congo (DRC) and disproportionately affects rural areas [1]. The DRC alone accounted for 13% of global malaria cases in 2023 [2]. The use of long-lasting insecticidal nets (LLINs) is considered the cornerstone of malaria prevention in the DRC. Nets are offered free of charge in mass distribution campaigns and routinely during antenatal consultations (ANC). One of the objectives of the national malaria strategy is that 80% of children younger than 5 years of age should sleep under a LLIN [3,4]. This level (80%) of protection corresponds to about seven deaths avoided per 1000 mosquito nets distributed [5].

According to the activity reports of the National Malaria Control Program (NMCP) of the DRC, an increase in the LLIN coverage rate was observed between 2014 and 2016. However, the same reports indicate an unprecedented increase in morbidity and mortality, attributed to malaria, in children under 5 years of age [3]. Several hypotheses could be put forward to explain this paradox, namely: (i) mortality from another source wrongly attributed to malaria, (ii) low use of LLINs, despite its increased distribution [6], and (iii) decreased effectiveness of LLINs due to vector resistance to pyrethroids, a class of insecticide used to impregnate nets. To address these issues, the NMCP has encouraged research on alternative tools including those that can manage *Anopheles* mosquito resistance, to optimize the effectiveness of pyrethroid-impregnated LLINs. In the western and eastern regions of Africa where vector resistance to pyrethroids is present, the results of several investigations have suspected that *An. gambiae* s.l. resistance to pyrethroids is the main factor behind the decline in the effectiveness of LLINs [7]. In 2016, WHO approved new insecticides or combinations of insecticides to optimize the effectiveness of conventional LLINs [8]. The so-called next-generation mosquito nets, such as those impregnated with pyrethroids and the synergist piperonyl butoxide (PBO), pyrethroid-chlorfenapyr ITNs, and pyrethroid-pyriproxyfen LLINs have emerged as new control tools [9]. PBO is known to have the property of inhibiting oxidases that are strongly involved in the metabolism of pyrethroids [10]. Bioassays conducted in the DRC showed a significant improvement in the mortality of resistant mosquitoes when tested on pyrethoids with PBO [11,12]. Transmission dynamics models indicate that even low levels of resistance would increase malaria incidence due to reduced mortality of *Anopheles* malaria vectors and decreased overall community protection [13]. Switching to PBO nets could prevent up to 0.5 clinical cases per person per year in some resistance scenarios [13]. Several studies have investigated the effectiveness of PBO LLINs in reducing malaria transmission and its epidemiological impact; however, the majority of these studies have been conducted in controlled laboratory settings. Some of these studies have demonstrated the PBO LLIN’s effectiveness on entomological indicators of malaria transmission in the context of pyrethroid vector resistance [14]. Studies conducted in experimental huts have shown that PBO LLINs are more effective than pyrethroid-only LLINs [15,16,17,18,19,20]. This finding was also supported by research focused on nets that have been used in the field [21,22].

The aim of the study was to assess the effectiveness of PBO-treated LLINs compared to standard pyrethroid-only nets, in the context of mosquito resistance to pyrethroids and their impact on malaria transmission under community conditions in the Democratic Republic of the Congo

## 2. Materials and Methods

### 2.1. Study Area

**Figure 1:**
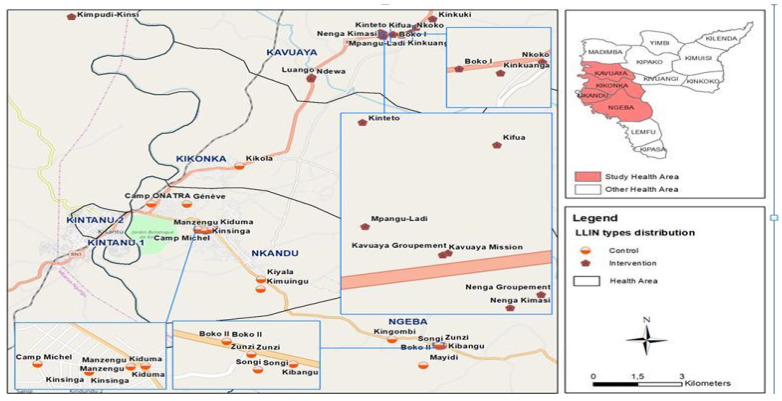
Location of study sites within Kisantu Health Zone, Kongo-Central, 2018.

This study was conducted in the Kisantu Health Zone (HZ), one of the HZs in Kongo-Central province, where malaria prevalence rates were higher than the national average according to the 2018 multiple indicator cluster survey in the DRC (MICS6) [23]. The study focused on 4 health areas (HA) out of the 15 that make up the Kisantu HZ: Kandu, Kikonka, Kandu, and Ngeba. These HAs had 100% data completeness in District Health Information Software 2 (DHIS2) for the first half of 2017, and the highest malaria frequencies in children under five. The mass LLIN distribution campaign took place in December 2017 in the four selected HAs (Figure 1).

### 2.2. Design

**Figure 2:**
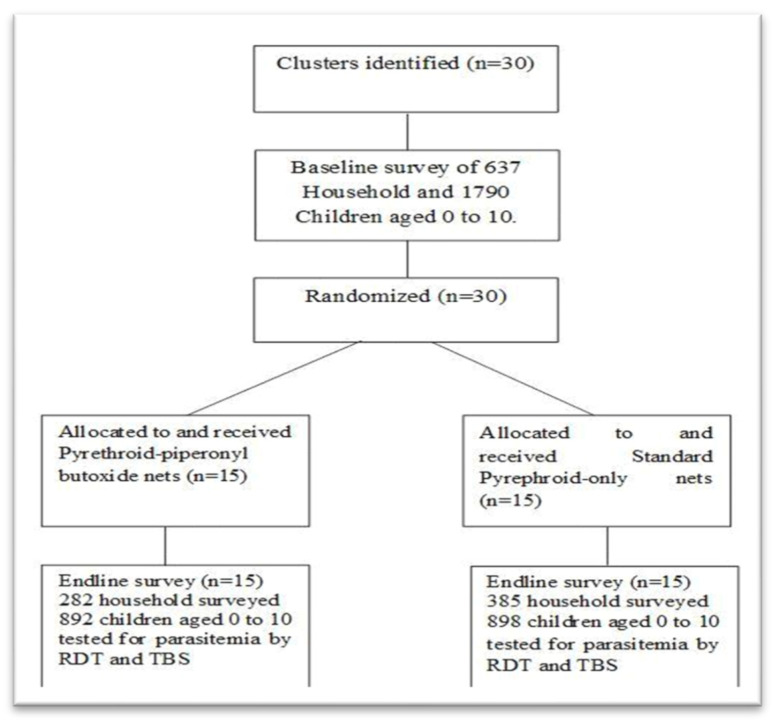
Overview of trial in the Kisantu Health Zone.

A quasi-experimental study was conducted to assess the effectiveness of pyrethroid PBO nets compared to standard pyrethroid-only nets for 12 months. To avoid contamination of the impregnated chemicals, the four HA were formed into two pairs taking into account their geographical proximity. Then the list of villages was drawn up and 30 villages were selected by simple random sampling out of the 72 villages in the 4 HAs, at a rate of 15 villages per pair of HAs (Figure 2). Assignment to the control group and the intervention group was made by applying the PBO implementation criterion, namely: having at least a malaria prevalence of 20%, and mosquito mortality in the bio-efficacy tests being ˂80% mortality [24]. In each village, a pilot survey was carried out and 60 children from age 0 to 10 were selected by systematic random sampling using sampling intervals.

### 2.3. Study Population and Sample Size

The sample size was calculated using Epi info 7.2.3.1, following the formula for calculating the sample size of cohort studies. The prevalence of malaria in the intervention group obtained during the baseline study was used (24.5%) as the proportion for the unexposed group. Given that a 25% reduction in this prevalence was expected, the proportion in the exposed group was 18.375%. The unexposed/exposed ratio was 1:1, with a power of 80% and a confidence threshold of 95%. This calculation resulted in a minimum sample size of 1472 children. The expected loss to follow-up was set at 15% (0.15) or 1472 × 0.15 = 220. The total sample size was 1472 + 220 = 1692 children, hence 846 children in 15 clusters per arm. As a result, 56 children were targeted for inclusion per cluster. In total, 1790 children (from 637 households) were enrolled in the study based on the following inclusion and exclusion criteria.

#### 2.3.1. Inclusion Criteria

Age equal to or less than 10 years.

Inhabitants of the selected villages.

#### 2.3.2. Exclusion Criteria

Previous known history of allergy to insecticide reported by parents.

Non-acceptance of the informed and written consent.

### 2.4. Data Collection and Study Procedures

#### 2.4.1. Case Surveillance

The children in the study were visited weekly at home by study staff for active case detection. When malaria symptoms were observed, Giemsa stained thick and thin blood films were examined under a microscope. Children who tested positive for malaria were treated according to the DRC national guidelines [25]. If a child was absent during a visit, a follow-up visit was planned. All minor or serious adverse events were recorded and managed in collaboration with the Clinical Pharmacology and Pharmacovigilance Unit of the University of Kinshasa. The results of these adverse events have already been published [26].

#### 2.4.2. Mosquito Net Distribution

From the 30 clusters, 15 were randomly assigned to receive PermaNet 3.0 (Batch number 102 9 17), which is made from a combination of deltamethrin and PBO LLINs. The roof of this LLIN consists of a knitted 100 denier monofilament polyethylene fiber blended with deltamethrin (4 g/kg, 122 mg/m^2^) and piperonyl butoxide (PBO) (25 g/kg). Its walls are knitted multifilament polyester (75 denier) fibers coated with deltamethrin (86 mg/m^2^). The remaining 15 clusters were assigned to receive Dawa Plus (Batch number 1356-B), with the following characteristics: 80 mg/m^2^ fiber polyester of 75 deniers, impregnated with deltamethrin.

#### 2.4.3. Mosquito Collection and Transmission Risk Determination

Anopheles mosquitoes were captured quarterly (for a total of five captures) in each of the 30 villages included in this study using human landing catches (HLC) and CDC Prokopack aspirators. HLC were performed from 6:00 p.m. to 6:00 a.m. to assess mosquito biting time, feeding behavior, and biting rates, and to monitor species composition and sporozoite rates. Trained residents collected adult mosquitoes over four consecutive nights in three different houses each night (a total of ninety houses per arm), with one person located indoors and another outdoors in each selected house. Collectors rotated indoors and outdoors every hour. The same houses were used every trimester.

CDC Prokopack aspirator collections were conducted from 6:00 a.m. to 9:00 a.m. in the same areas as the HLC (in different houses to the HLC) to estimate the indoor resting density of mosquito species. Female *Anopheles* were classified according to the four abdominal stages (unfed, fed, half-gravid, and gravid).

All *Anopheles* mosquitoes collected by HLC and CDC Prokopack aspirators were morphologically identified to species in the field and cross-checked by DRC National Institute of Biomedical Research (INRB) entomologists either in the field or in Kinshasa (depending on the supervision schedule). *Anopheles* mosquitoes were preserved in 1.5 mL Eppendorf tubes with silica gel for further analysis. Circumsporozoite surface protein of *Plasmodium* sporozoites was detected by enzyme-linked immunosorbent assay (ELISA) in *Anopheles gambiae* s.l. and *Anopheles funestus* in Kinshasa in INRB. A randomly selected subset of *Anopheles gambiae* mosquitoes were assessed for the presence of the *kdr* mutation using PCR, in the CDC entomological laboratory in Atlanta, Georgia, USA [15].

#### 2.4.4. Durability Surveys: Physical Integrity and Bio-Efficacy of LLINs

LLINs were sampled twice every six months (six and twelve months after the LLIN mass distribution) in collaboration with community health workers. They were collected from one house (different houses for each sampling) in each of the villages, totaling 60 LLINs (30 PermaNet 3.0, 30 Dawa Plus). All collected nets were replaced with new ones of the same type. The number and size of holes were assessed according to standard protocols outlined by WHO (2011) [27].

Four pieces of netting, each measuring 30 cm by 30 cm, were cut from the sides of each LLIN (Dawa Plus and PermaNet 3.0), and an additional four pieces were cut from the roof of each PermaNet 3.0. For the bioassay, one piece from the sides of a Dawa Plus net and two pieces from a PermaNet 3.0 net (one from the side and one from the roof, randomly chosen) were used. These netting pieces were sent to the “Centre de Recherche Entomologique de Cotonou” (CREC, Benin) for assessment using the standard WHO bioassay cone test procedure [27]. In total, five mosquitoes were exposed to each netting piece. For the standard pyrethroid nets (Dawa Plus), five mosquitoes were tested on the side, while for the nets with PBO (PermaNet 3.0), five mosquitoes were tested on the side and 20 on the roof of the net. After exposure, mosquitoes were transferred to clean paper cups and provided with a sugar solution, and knockdown was recorded at 3, 10, 30, and 60 min after the start of the assay. Mortality was recorded after a 24 h holding period. LLIN samples were also shipped to CDC/Atlanta for further cone bioassay testing using pyrethroid-resistant laboratory strains and to measure chemical content [28].

### 2.5. Operational Definition of Key Study Variables

Malaria infection was defined as the presence of parasites on thick blood smears.

The following formulas were used to calculate entomological indicators:

- The sporozoite rate = (total CSP ELISA positive/total number tested) × 100;
- Human biting rate (HBR) = total # of each Anopheles species collected by HLCs during a specific period/total number of trap nights;
- Nightly EIR = nightly HBR × sporozoite rate;
- Monthly EIR = nightly mean EIR × number of nights in the month;
- Physical net survival: the proportion of cohort nets received from the LLIN campaign still in serviceable physical condition (WHO 2013 estimating functional of LLIN).

Net durability variables.

Net attrition rate due to wear and tear: the proportion of originally received nets that were lost due to wear and tear at the time of assessment.

Net integrity was measured first by the proportionate hole index (pHI) as recommended by WHO [28].

Median net survival was defined as the time in years until 50% of the originally distributed LLIN were no longer serviceable.

#### 2.5.1. Outcomes

The primary outcome was the incidence of malaria infection in children included in the study, measured monthly over 12 months of follow-up after the intervention (distribution of LLINs). Secondarily, a comparison of malaria incidence was also made monthly between the two arms.

The outcome of malaria transmission was the entomological outcome, which included the inoculation rate, sporozoite rate, and genetic resistance mechanisms in mosquitoes over a period of 3, 6, 9, and 12 months. The insecticidal effectiveness was assessed based on the bioassay.

#### 2.5.2. Randomization and Masking

In November 2017, a selection of the 30 clusters using Stat version 10.0 made it possible to randomize the villages into two groups (Figure 1 and Figure 2). Each group had an equal number of clusters (15) and depending on the type of net allocated per group, the PBO net arm was considered as the intervention group and the standard type as the control.

The bales of nets had the same shape, size, and color of packaging. Only the principal investigator and the zone chief medical officer knew the type of nets. Users and campaign team members were not informed. Thick, thin blood smears were coded to the identity and status of the participants’ intervention. Only the principal investigator knew these identities.

### 2.6. Data Analysis

The data collected were entered using Epi-Info 7 software. To test if the malaria incidence and mosquito densities differed significantly between control and intervention villages, R 2024.12.0 software was used (multilevel mixed-effects linear regression model). The exploratory variables were time (‘month’) and ‘treatment’. GAMMs were used because we expected a nonlinear response for the time variable. A smooth term was used for the variable month to correct for non-linear temporal correlation. “Village” was included in the model as a random effect. The response variable was malaria incidence or mosquito density per village which was modeled assuming a Poisson distribution. The optimal amount of smoothing was determined by cross-validation using the built-in function of R-package gamm4.

Vector density and entomological inoculation rate were analyzed with negative binomial regression, after adjusting for the baseline. The entomological inoculation rate was estimated as the mean number of sporozoite-infected *Anopheles* per house per night [29], and weighted to account for the proportion of collected *Anopheles* processed for sporozoites.

The physical integrity of campaign LLINs was assessed in accordance with WHO guidelines, with the number of holes of size 0.5–2 cm diameter (size 1), 2–10 cm diameter (size 2), 10–25 cm diameter (size 3), and >25 cm diameter (size 4) recorded for each net following examination by the team in a well-lit location. Data from the LLIN hole assessment were transformed into the proportionate hole index (pHI) for each LLIN using the following standard weights defined by WHO:

pHI = Number of size 1 hole + (No. of size 2 holes × 23) + (No. of size 3 holes × 196) + (No. of size 4 holes × 576).

Based on the pHI value, LLINs were categorized as “good”, “serviceable” or “torn” as defined below. Note that “good” is a subset of all “serviceable” LLINs.

Good: pHI < 64 (corresponding to a total hole surface area < 0.01 m^2^)

Serviceable: pHI < = 642 (total hole surface area <= 0.1 m^2^)

Torn: pHI > 642 (total hole surface area > 0.1 m^2^)

The outcomes of insecticidal effectiveness were based on the bioassay results performed by CREC in Benin. The 60-min knockdown (KD60) and the 24-h mortality rate (mortality) were measured. The two variables from these tests were combined into the following outcome measures:

Optimal effectiveness: KD60 ≥ 95% or mortality ≥ 80%.

Minimal effectiveness: KD60 ≥ 75% or mortality ≥ 50%.

### 2.7. Ethical Considerations

The study received approval (ESP/CE/061/2016) from the ethics committee of the School of Public Health of the University of Kinshasa, DRC. After a detailed explanation of the purpose of the study, written informed consent was obtained from parents or guardians. All febrile participants received an antipyretic and those with a positive RDT or thick slide were treated according to national treatment guidelines.

This study was conducted in accordance with good clinical practice and the Declaration of Helsinki. LLINs were provided free of charge, and the costs of care for positive patients were covered by the study. This trial was registered in ClinicalTrials.gov #NCT03289663 (https://clinicaltrials.gov/study/NCT03289663, accessed on 21 December 2024).

## 3. Results

### 3.1. General Characteristics of Inclusion

In total, 637 households were enrolled and 1790 children aged from 0 to 10 years old were tested for malaria. Participants under 5 years of age represented 51.9% of all participants. Among those included in the present study, 51% were male (*p* = 0.959), resulting in a male-to-female sex ratio of 1.057. The frequencies of the positive blood smear were 24.5% and 5.3%, respectively, in the intervention group and the control group. More details on socio-demographic information on the study population are provided in Table 1.

### 3.2. Incidence of Malaria

**Figure 3:**
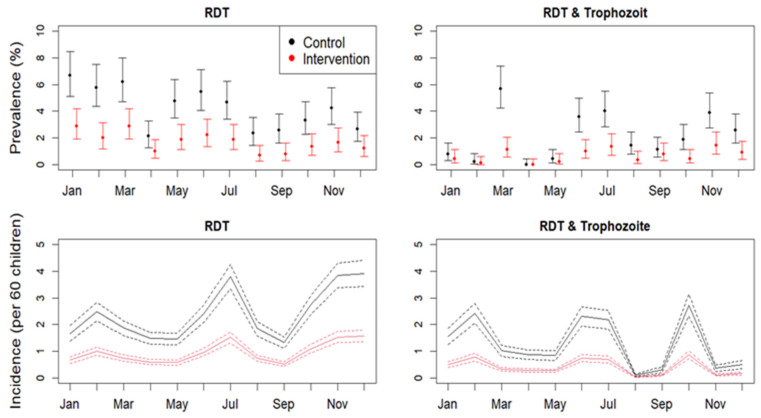
Mean malaria incidence for the control versus intervention in Kisantu Health Zone, Kongo-Central, 2018.

There was a significant non-linear effect of time (months) on the malaria thick blood smear (TBS) incidence (edf = 8.3, F = 14.2, *p* < 0.0001). The incidence was higher in the months of January–March, May–June, and November. It decreased in April, August-September, and December. Although this pattern was found for both the control and intervention villages, we observed that the prevalence in the control villages was overall higher (estimate = 0.9, std error = 0.12, t value = 7.5, *p* < 0.001) (Figure 3).

### 3.3. Entomological Impact

**Figure 4:**
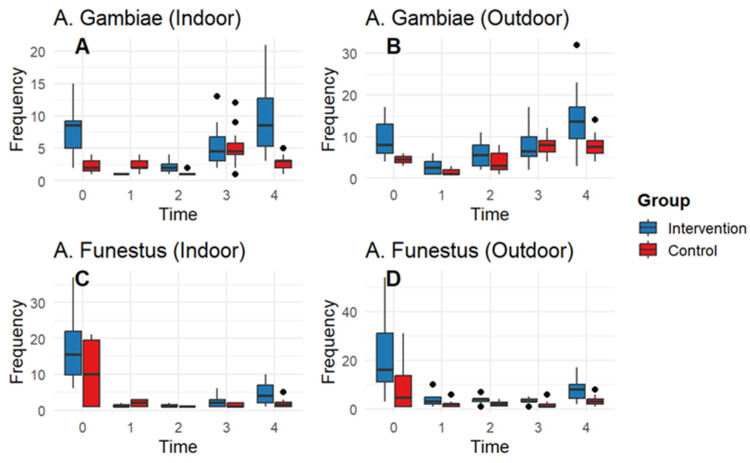
Impact of the mosquito net on the *Anopheles* population in Kisantu Health Zone, Kongo-Central, 2018.

The GAMM generally fitted the data well (R^2^ = 0.3–0.5), and no major deviations from the functional form or dependencies in the residual data were observed. The malaria incidence in Kisantu showed a clear seasonal pattern (edf = 8.9, chisq = 119.3, *p* < 0.0001). This pattern was characterized by peaks in June–July and December–February, one–two months after the end of the rainy seasons. Incidences of malaria was significantly lower in the intervention group throughout the year of the study with 2–4 individuals per cohort per month (est = −0.922, z-value = −10.05, *p* < 0.001 *). Similarly, we observed a significant non-linear effect of time on the density of both *An. funestus* versus *An. gambiae* indoor (edf = 1.97, chisq = 264.1, *p* < 0.0001 vs. edf = 1.97, chisq = 52.5, *p* < 0.0001) and outdoor (edf = 1.97; chisq = 334; *p* < 0.0001 vs. edf = 1.98, chisq = 132, *p* < 0.001) (Figure 4 and Table 2). Densities decreased after the first month of the intervention but were already very low in the control group. The densities increase after time point 2 in both the intervention and control groups. The mosquito densities were in general lower in the control versus the intervention group, also at the start of the experiment.

The biting rates of *Anopheles gambiae* s.l. and *Anopheles funestus* s.l. in the control group varied with time, from T1 to T4, from 0.021 to 0.80 and 0.22 to 0.36 bites per person per night respectively (Table 3). In the intervention group it varied also for *Anopheles gambiae* s.l. from 0.20 to 1.83, while for *Anopheles funestus* s.l., it was 0.30 in Trimester (T) 1, 0.22 in T2, 0.38 in T3 and 0.95 in T4. The inoculation rate, however, was 0.05 in T4 for the intervention group while for the control group, it was zero in the same period. The entomological inoculation rate was zero for *Anopheles funestus* s.l. in both the intervention and control groups (Table 3).

The female Anopheles were classified according to the four abdominal stages (unfed, fed, half-gravid, and gravid), respectively, 1%, 67%, 13%, 19% in the intervention zone and 2%, 71%, 15%, 12% in the control zone (Supplementary File).

### 3.4. LLIN Physical Integrity, WHO Cone Bio-Assays and LLIN Chemical Content

From the physical durability point of view, 5/359 LLINs were considered to be damaged in the control group and this was statistically significant (*p* = 0.009).

Regarding the bio-efficacy, at the 12-month timepoint, the 60 min knockdown was 53.8% (95% CI: 51.9–55.5) after 12 months for the intervention group versus 46.8% (95% CI: 44.7–49.0) for the control group, while the 24 h mortality was 46.7% (95% CI: 44.7–49.0) for the intervention group versus 22.5% (95% CI: 20.2–24.7) for the control group. This showed a significant difference (*p* = 0.001) (Table 4). For the chemical content of the nets, a 6-month deltamethrin loss was, respectively, for the intervention and control group 69.1% (95%CI: 64.4–73.1) versus 29.3% (95% CI: 24.0–35.1), and at 12 months, 52.8% (95% CI: 44.4–60.0) versus 37.4% (95% CI: 32.9–42.2) (Table 4).

## 4. Discussion

This trial evaluated the efficacy of PBO LLINs versus pyrethroid-only LLINs in the Kisantu HZ, where *Anopheles* mosquitoes have shown resistance to pyrethroids, as demonstrated earlier [15].

The arm with PBO LLINs resulted in reduced malaria incidence over 12 months compared to the arm with pyrethroid LLINs alone. Children in the intervention group were less likely to develop malaria after LLIN distribution than children in the control arm over 12 months.

Entomological inoculation was reduced in both arms, but more pronounced in the intervention arm, resulting in a vector population reduction in density and longevity. The additional protection provided by LLINs with piperonyl butoxide was variable during the 12 months of observation; this could be explained by the fact that malaria has seasonal transmission. Over the 12 months of use, from the physical, chemical, and conical points of view, high durability characterized the intervention group, unlike the control group. However, it experienced a 70% insecticide loss.

The clinical incidence of malaria in children has almost halved, with a reduction in entomological inoculation rates. The specific estimate of malaria incidence and entomological inoculation rate remained lower in the LLIN piperonyl butoxide group compared to the control group over time. Piperonyl butoxide LLINs were more efficient compared to pyrethroid-LLINs alone.

However, several authors have already presented data on the effectiveness of these LLINs, reporting higher mortality in resistant *Anopheles gambiae* [16,18,19,20,30] and *Anopheles funestus,* compared to LLIN pyrethroids alone [31]. According to experimental studies on huts, intensive net washing did not significantly reduce effectiveness in these studies. The substantial reduction in vector density and the maintenance of the effect on malaria outcomes within 12 months confirm the lasting efficacy of deltamethrin+PBO. This efficacy was achieved despite the decrease in the concentration of deltamethrin to more than 40%, compared to the manufacturer’s declaration within 12 months. Efficacy was marked by malaria reduction (low incidence and low entomological inoculation rate) in the first trimester of observation. However, for this study with a large loss of the insecticide, a limit is observed by referring to the WHO recommendation, stipulating a continuous use of the net within 3 years. This loss of insecticide calls into question the usual interval between LLIN distribution campaigns and requires the revision of the frequency of replacement of these LLINs. Mansiangi and colleagues hypothesized that the interval between campaigns is two years for the possible effectiveness of LLIN [32]. According to this study, both models for a 12-month observation are below the threshold set by the WHO. Further studies could be the subject of future guidelines to determine or improve the duration of LLIN recycling.

Deltamethrin+PBO LLINs showed additional efficacy compared to pyrethroid LLIN only over a shorter period (12 months) than in earlier RCT (21 to 25 months) [33]. In an earlier RCT in Tanzania, the use of the same brand of piperonyl butoxide LLIN was 50% in the second year, which may explain why efficacy was maintained longer in this study than in the current study, for which the piperonyl butoxide used decreased more rapidly (to about 30%) after 2 years [34,35]. Current malaria control strategies are based on an assumed effective lifespan of 3 years for insecticide-treated nets. This combination is far from satisfying this strategy, by the rapid loss of the concentration of the insecticide. This has been described in other studies such as in northwestern Tanzania and Kenya [36,37]. The use of piperonyl butoxide LLINs was also lower than that of LLINs of the study in the other groups in this study. Of the piperonyl butoxide LLINs still in service after 12 months, the proportion of nets that were torn was less than that in the control group.

The combined evaluation of our epidemiological trial, entomological outcomes, and LLIN sustainability significantly advances the conclusive evidence on LLIN choices taking into account the type of active resistance for malaria control [38,39].

Nevertheless, limitations remain. First, the rapid decrease in the use of LLIN in studies within a context of high overall net use may partly explain the relative lack of efficacy of piperonyl butoxide LLINs over 12 months compared to pyrethroid-only ITNs. Second, the cluster size of the buffer zones. Although they can be maximized due to the study, they may not have completely prevented contamination of effects with the intervention group. The present study did not resort to a randomized trial for the simple reason that it was difficult to do so on an individual scale. This is due to the difficulty of controlling contamination but also from an entomological point of view in order to avoid the repulsion of mosquitoes. The socio-demographic differences between the two arms were taken into account during the statistical analyses by comparing the proportional differences using the software. Third, whether piperonyl butoxide LLINs maintain their apparent efficacy during a third year of use, knowing that the presence of *Anopheles* with high genetic resistance could be a major challenge to maintain this efficacy. This study represents the first in the DRC, to our knowledge, with demonstrable effectiveness in malaria control. Unlike all other insecticides used for malaria control, piperonyl butoxide has a mode of action on a known metabolic resistance in mosquitoes. However, caution is exercised in scaling up a mass distribution of pyrethroid-based LLINs alone, because of the spread of resistance to pyrethroids. Considering that WHO globally has developed a management plan for insecticide resistance in malaria vectors after resistance to pyrethroids has already been widespread, the challenge would be to preserve the effectiveness of piperonyl butoxide by developing resistance management strategies, substantial gains could be made with wider deployment of these nets. However, manufacturers need to improve the sustainability of LLIN textiles, encouraged by global sourcing approaches that focus not only on LLIN prices but also on performance and sustainability. Further studies based on LLINs capable of taking into account both metabolic and genetic resistance in vectors are needed to optimize the malaria control strategy.

## 5. Conclusions

The nets treated with the combination of PBO and deltamethrin tend to be somewhat more effective for malaria control under community conditions in the DRC, but this effectiveness does not last for a long duration. A review of the distribution cycle is desirable, along with post-campaign monitoring in order to preserve the durability of the mosquito net.

## Figures and Tables

**Table 1:** General characteristics of the study population in the two arms of the study in Kisantu Health Zone, Kongo-Central Province, 2018.

Variables	All	Control	Intervention
Sex			
Female	870 (48.6)	437 (48.7)	433 (48.5)
Male	920 (51.4)	461 (51.3)	459 (51.4)
Age			
<5 years	925 (51.9)	440 (49.0)	485 (54.8)
≥5 years	865 (48.3)	458 (51.0)	407 (45.6)
Type of roof			
Thatch/wood	258 (14.4)	12 (1.3)	246 (27.6)
Metal sheet	791 (44.2)	570 (63.5)	221 (24.8)
Straw	741 (41.4)	316 (35.2)	425 (47.7)
Type of wall			
Clay with pillar	350 (19.9)	31 (3.5)	319 (35.8)
Cement brick/baked	1393 (77.8)	865 (96.3)	528 (58.2)
Straw	47 (2.6)	2 (0.2)	45 (5.0)
Type of floor			
Cement/tiled	687 (38.4)	577 (64.3)	110 (12.3)
Ground/bamboo	1103 (61.6)	321 (35.8)	782 (87.8)
Wasting			
Moderate/severe	1211 (67.7)	631 (70.3)	580 (65.0)
Normal/mild	268 (14.9)	134 (14.9)	134 (15.0)
Over	311 (17.4)	133 (14.8)	178 (19.9)
Stunting			
Moderate/severe	608 (33.9)	312 (34.7)	296 (33.2)
Normal/mild	967 (54.0)	492 (54.8)	475 (53.3)
Over	215 (12.0)	94 (10.5)	121 (13.6)
Underweight			
Moderate/severe	461 (25.8)	223 (24.8)	238 (26.7)
Normal/mild	1050 (58.7)	539 (60.0)	511 (57.3)
Over	279 (15.6)	136 (15.1)	143 (16.0)
Thick Blood Smear			
No	1524 (85.1)	851 (94.77)	673 (75.5)
Yes	266 (14.8)	47 (5.3)	219 (24.5)

**Table 2:** Impact of mosquitoes in the model.

System	Time	Intervention
*An. gambiae*_out	edf = 1.98, chisq = 132 *p* < 0.001	est = −0.46; std error =0.007; z value = −613; *p* < 0.0001
*An. gambiae*_in	edf = 1.97, chisq = 52.5, *p* < 0.0001	est = −0.69; std error= 0.09; z value = −71; *p* < 0.0001
*An. funestus*_in	edf = 1.97, chisq = 264.1, *p* < 0.0001	est = −0.629; std error = 0.1135; z value = −5.5; *p* < 0.0001
*An. funestus*_out	edf = 1.97, chisq = 334; *p* < 0.0001	est = −0.7392; std error =0.09774; z value = −7.5; *p* < 0.0001

**Table 3:** Entomological Inoculation Rate of *Anopheles gambiae* s.l./*Anopheles funestus* s.l. in Kisantu Health Zone, Kongo-Central Province, 2018.

Arms	Trimester	Total An Collected HLC	HLC Trap-Nights	HBR/Night	Number of An Tested	Pf Sporozoite Rate	EIR/ Night	EIR/ Month
*Anopheles gambiae sl.*								
Control	baseline	16	180	0.08	16	1	0.08	2.4
	T1	38	180	0.21	44	0.05	0.01	0.3
	T2	41	180	0.23	50	0	0	0.0
	T3	181	180	1.00	97	0.03	0.03	0.9
	T4	144	180	0.80	99	0.01	0.00	0.0
Intervention	baseline	222	180	1.23	50	4	4.92	147.6
	T1	37	180	0.20	33	0.06	0.01	0.3
	T2	85	180	0.47	85	0.40	0.18	5.4
	T3	184	180	1.02	93	0.03	0.03	0.9
	T4	330	180	1.83	100	0.03	0.05	1.5
*Anopheles funestus sl.*								
Control	baseline	83	180	0.46	39	2	0.92	27.6
	T1	39	180	0.22	42	0.02	0.00	0.0
	T2	15	180	0.08	17	0	0.00	0.0
	T3	32	180	0.17	1	0	0.00	0.0
	T4	65	180	0.36	1	0	0.00	0.0
Intervention	baseline	489	180	2.71	50	0	0.00	0.0
	T1	54	180	0.30	55	0	0.00	0.0
	T2	40	180	0.22	40	0	0.00	0.0
	T3	69	180	0.38	1	0	0.00	0.0
	T4	171	180	0.95	1	0	0.00	0.0

**Table 4:** Durability, physical integrity, cone bioassay, and chemistry dosage of LLINs in Kisantu Health Zone, Kongo-Central Province, 2018.

Variable	6 Months			12 Months		
	Control	Intervention	*p*	Control	Intervention	*p*
1. Physical condition (pHI)	N = 364	N = 369		N = 364	N = 368	
Good (0–64)	359	369	0.009	359	368	0.009
Damaged (65–642)	5	0		5	0	
Torn (>642)						
Serviceable (0–642)						
Median pHI if any hole IQR	2 (0)	2 (0)		2 (0)	2 (0)	
2. bio-assays (WHO)	N = 30	N = 30		N = 30	N = 30	
Knockdown 60 min						0.001
Mean (95% CI)				46.81 (44.69–49.00)	53.74 (51.97–55.45)	
Median (IQR)				48.0 (41–51)	53.00 (52–57)	
Mortality 24 h						0.001
Mean (95% CI)				22.47 (20.19–24.69)	46.74 (44.36–48.99)	
Median (IQR)				23 (18–26)	47 (45–51)	
Optimal effectiveness				38	60	
Minimal effectiveness				8	26	
3. chemistry	N = 30	N = 30		N = 30	N = 30	
Mean (95% CI)	29.32 (24.01–35.06)	69.14 (64–42-73.10)	0.000	37.39 (32.93–42.20)	52.78 (44.43–60.01)	0.001
Median (IQR)	31.35 (18.15–43.10)	72.25 (61.15–77.45)		37.95 (30.50–43.60)	59.60 (41.50–67.45)	
SD	16.75 (13.29–19.24)	12.18 (7.87–16.07)		12.20 (9.46–14.44)	19.21 (12.30–23.15)	
Roof PermaNet 3.0 (mean)					121.9	

## Data Availability

The authors confirm that all data supporting the findings are fully accessible and available without any restrictions. All relevant data supporting the findings of this study are included in the paper and its Supporting Information Files.

## Supplementary Materials

The following supporting information can be downloaded at: https://www.mdpi.com/article/10.3390/tropicalmed10060172/s1, Table S1: distribution of Anopheles females according to abdominal stages in the sites.
