# Supplementary material for: Effectiveness of Pyrethroid-Piperonyl Butoxide Nets Versus Standard Pyrethroid-Only Nets in Preventing Malaria in Children Under 10 Years Living in Kisantu Health Zone, Democratic Republic of the Congo
Source: Trop Med Infect Dis. 2025 Jun 18;10(6):172. doi: 10.3390/tropicalmed10060172 (PMC12197424; doi:10.3390/tropicalmed10060172)
Supplement: Supplementary file 1 [file tropicalmed-10-00172-s001.zip › tropicalmed-3518916-supplementary.pdf]

Table S1: Distribution of Anopheles females according to abdominal stages in the sites.

**Site**	**Unfed**	**Fed**	**Semi-gravid**	**Gravid**
Intervention	1%	67%	13%	19%
Control	2%	78%	8%	12%
